# Web-based dietary assessment and advice helps inflammatory bowel disease patients to improve their diet quality

**DOI:** 10.1017/S0007114522001064

**Published:** 2023-01-28

**Authors:** Carlijn R. Lamers, Liselot W. van Erp, Anne I. Slotegraaf, Marcel J. M. Groenen, Nicole M. de Roos, Peter J. Wahab, Ben J. M. Witteman

**Affiliations:** 1 Department of Gastroenterology and Hepatology, Hospital Gelderse Vallei, Ede, The Netherlands; 2 Division of Human Nutrition and Health, Wageningen University & Research (WUR), Wageningen, The Netherlands; 3 Crohn & Colitis Centre, Department of Gastroenterology and Hepatology, Rijnstate Hospital, Arnhem, The Netherlands; 4 Nutrition & Healthcare Alliance, Ede, The Netherlands

**Keywords:** Dietary assessment, Crohn’s disease, Ulcerative colitis, Quality of life

## Abstract

Time to evaluate diet quality and give dietary advice is limited in clinical inflammatory bowel disease (IBD) practice. The *Eetscore* is a web-based tool that assesses diet quality according to the Dutch dietary guidelines and provides personalised dietary advice. We aimed to assess diet quality of IBD patients using the *Eetscore* and to study changes in diet quality, health-related quality of life (HRQoL) and clinical disease activity over time. A prospective cohort study was performed in 195 adult IBD patients. Participants were invited to fill out questionnaires (*Eetscore*-FFQ, short Inflammatory Bowel Disease Questionnaire and Patient Harvey Bradshaw Index/Patient Simple Clinical Colitis Activity Index) at baseline and after 1 and 4 months. The *Eetscore* calculates diet quality based on sixteen food components (ten points per component, total score 0–160; the higher the better) and provides dietary advice per component based on the assessment. At baseline, mean diet quality was 98 (sd 19). Diet quality was positively associated with age, female sex and level of education. Component scores were highest for red meat, wholegrain products and sweetened beverages, and lowest for legumes, nuts and processed meat. Over time, diet quality increased to 107 (sd 21) at 4 months (*P* < 0·001). Each ten-point improvement in diet quality was associated with an increase in HRQoL (*β* = 0·4 (95 % CI (0·02, 0·7), *P* = 0·04). Clinical disease activity did not change. In conclusion, diet quality of IBD patients significantly improved following personalised dietary advice of the *Eetscore*. Improvement of diet quality was associated with a slight improvement in HRQoL. The *Eetscore* is a practical and useful tool to monitor and support a healthy diet in IBD patients.

Crohn’s disease and ulcerative colitis are chronic inflammatory bowel disease (IBD) with a complex aetiology including genetic, microbial, immune and environmental factors^(1)^. Increasing evidence suggests that a person’s diet is an important environmental factor contributing to the development of IBD and its disease course^(2)^. Dietary components may influence the pathogenesis of IBD through their effects on the microbiome, mucosal barrier and immune response^(2)^. Although many IBD patients believe diet affects the course of their disease and is at least as important as medication in their IBD treatment, evidence supporting beneficial effects of specific dietary patterns in adult IBD patients is limited^(3,4)^.

Recently, the International Organization for the Study of Inflammatory Bowel Diseases attempted to establish a consensus document stating which foods may be either beneficial, harmful or safe to consume^(5)^. Due to lack of evidence, recommendations were limited but in line with the general guidelines for a healthy diet, such as to consume more fruit, vegetables and *n*-3 fatty acids, and to consume less saturated, trans, and dairy fat and red and processed meat^(5)^. Therefore, general dietary guidelines may also be recommended to patients with IBD.

In the Netherlands, the composition of a healthy diet is described by the Health Council in the Dutch dietary guidelines^(6,7)^. These guidelines advise to adopt a more plant-based and less animal-based dietary pattern by eating sufficient fruit, vegetables and wholegrain products, moderating the intake of meat, varying between fish, legumes, nuts and eggs and taking sufficient dairy products and fluids. In clinical practice, IBD patients are interested in the beneficial effects of diet on the course of their disease. However, time is limited to assess a patients’ food intake and to support a healthy diet. It would be helpful if patients could assess their own diet quality and receive personal advice outside the consultation room.

A web-based tool that may be used to assess diet quality and to support a healthy diet is the *Eetscore* (‘Eatscore’)^(8)^. This is a validated tool to assess adherence to the Dutch dietary guidelines, that is, diet quality^(9)^. Based on the assessment, the *Eetscore* provides personalised dietary advice to eat healthier and more in line with the Dutch dietary guidelines. It can also be used to monitor diet quality over time. So far, the *Eetscore* has only been used in healthy populations and CVD patients, but not in IBD patients^(10,11)^.

We used the *Eetscore* tool to assess diet quality of IBD patients, to evaluate if diet quality improves after personalised dietary advice and to study factors associated with diet quality in IBD patients. In addition, we aimed to evaluate whether changes in diet quality resulted in changes in health-related quality of life (HRQoL) and clinical disease activity. We also evaluated the experiences of IBD patients with the *Eetscore* tool.

## Materials and methods

### Study population and design

A prospective cohort study was performed in adult IBD patients. Participants were recruited between October 2020 and February 2021 from the outpatient clinics of Hospital Gelderse Vallei in Ede and Rijnstate Hospital in Arnhem, the Netherlands. Both are secondary care referral centres, with IBD-dedicated gastroenterologists and nurses, and are collaborative partners of the Nutrition & Healthcare Alliance, an expert centre in nutrition and healthcare. Inclusion criteria were diagnosis of Crohn’s disease, ulcerative colitis or IBD-unclassified, 18 years of age or older, ability to read and understand the Dutch language and access to an e-mail address and a device to complete the online questionnaires. Exclusion criteria were current dietary counselling by a dietician or lifestyle coach, allergy for nuts, peanuts, fish or cows’ milk protein (to protect them from potential harmful recommendations to consume these products), adherence to a vegan lifestyle (to avoid invalid dietary assessment as the *Eetscore* does not assess plant-based alternatives) and participation in another intervention study. This study was approved by the Medical Ethical Committee of Wageningen University (METC nr. 20/16) and conducted in accordance with the Declaration of Helsinki and registered at trialregister.nl (NL8784). All participants provided written informed consent.

### Data collection

After the informed consent form was signed, participants were invited via e-mail to complete questionnaires assessing diet quality, HRQoL and clinical disease activity. Participants who completed the baseline assessment were invited to complete the questionnaires again after 1 and 4 months. These time points were chosen to assess short-term changes in diet quality as well as to evaluate effects at longer term. At baseline, participants were also asked about their level of education, smoking behaviour, dietary restrictions, and height and weight. At 4 months, participants were also invited to complete an evaluation about the *Eetscore*. Non-responders were reminded 5, 10 and 15 d after each invitation.

Relevant participant and disease characteristics were retrieved from medical records, including age, sex, diagnosis, disease duration, disease phenotype according to the Montreal classification, extra-intestinal manifestations, IBD medication used and IBD-related surgeries. The highest treatment step was documented, classified as 5-aminosalicylic acids, steroids, immunomodulators and biologics.

#### Eetscore

The *Eetscore* is a validated web-based tool that consists of: (1) a short FFQ assessing food intake (*Eetscore*-FFQ), (2) a diet quality score calculated using the Dutch Healthy Diet 2015 (DHD15)-index and (3) personalised dietary advice based on the assessment^(8–10)^.

The *Eetscore*-FFQ assesses intake of the following sixteen components: vegetables, fruit, wholegrain products, legumes, nuts, dairy products, fish, tea, fats and oils, coffee, red meat, processed meat, sweetened beverages and fruit juices, alcohol, salt and unhealthy choices. Participants reported their intake frequency of each component during the past month (‘never’ to ‘every day’) together with portion size (standard and natural portions (e.g. piece of fruit) or commonly used household measures (e.g. spoons or cups). It takes approximately 10–15 min to complete the *Eetscore*-FFQ.

The DHD15-index is calculated based on a person’s food intake as assessed by the *Eetscore*-FFQ. The criteria to calculate the DHD15-index have been described in detail elsewhere and are summarised in Table 1 ^(9)^. Components are categorised into five types: adequacy, ratio, optimum, qualitative and moderation component. Scores range from 0 to 10 points with cut-offs for each component based on the dietary guidelines (Table 1). In the total score, all sixteen components are combined, resulting in a score range of 0–160. Higher scores indicate better adherence to Dutch dietary guidelines.

**Table 1.** Cut-off and threshold values for calculation of the sixteen components

	Component	Component type*	Dutch dietary guidelines 2015	Minimum score (=0 points)	Maximum score (=10 points)
1.	Vegetables	A	Eat at least 200 g of vegetables daily	0 g/d	≥200 g/d
2.	Fruit	A	Eat at least 200 g of fruit daily	0 g/d	≥200 g/d
3.	Wholegrain products	A	Eat at least 90 g of wholegrain products daily	0 g/d	≥90 g/d
		R	Replace refined cereal products by wholegrain products	No consumption of wholegrain products or ratio of whole grains to refined grains ≤0·7	No consumption of refined products or ratio of whole grains to refined grains ≥ 11
4.	Legumes	A	Eat legumes daily	0 g/d	≥10 g/d
5.	Nuts	A	Eat at least 15 g of unsalted nuts daily	0 g/d	≥15 g/d
6.	Dairy products	O	Eat a few portions of dairy products daily, including milk or yogurt	0 g/d or ≥ 750 g/d	300–450 g/d
7.	Fish	A	Eat one serving of fish weekly, preferably oily fish	0 g/d	≥15 g/d
8.	Tea	A	Drink three cups of black or green tea daily	0 g/d	≥450 ml/d
9.	Fats and oils	R	Replace butter, hard margarines and cooking fats by soft margarines, liquid cooking fats and vegetable oils	No consumption of soft margarines, liquid cooking fats and vegetable oils or ratio of liquid cooking fats to solid cooking fats ≤ 0·6	No consumption of butter, hard margarines and cooking fats or ratio of liquid cooking fats to solid cooking fats ≥ 13
10.	Coffee	Q	Replace unfiltered coffee by filtered coffee	Any consumption of unfiltered coffee	Consumption of only filtered coffee OR No coffee consumption
11.	Red meat	M	Limit consumption of red meat	≥100 g/d	≤45 g/d
12.	Processed meat	M	Limit consumption of processed meat	≥50 g/d	0 g/d
13.	Sweetened beverages and fruit juices	M	Limit consumption of sweetened beverages and fruit juices	≥250 g/d	0 g/d
14.	Alcohol	M	If alcohol is consumed at all, intake should be limited to one Dutch unit (10 g ethanol) daily	Women: ≥ 20 g ethanol/d Men: ≥ 30 g ethanol/d	Women: ≤ 0 g ethanol/d Men: ≤ 0 g ethanol/d
15.	Salt	M	Limit consumption of table salt to 6 g daily	≥3·8 g Na/d	≤1·9 g Na/d
16.	Unhealthy choices†	M	Limit consumption of unhealthy day and week choices	>7 choices/week	≤3 choices/week

* A, adequacy component (minimum consumption); R, ratio component (replace products by more healthy alternatives); O, optimum component (optimal consumption range); Q, qualitative component (choose healthier option); M, moderation component (limit consumption).

† For example: sweet spreads, cakes, cookies, chips or pretzels, savoury snacks and sauces.

The *Eetscore* tool provides personalised dietary advice every time the *Eetscore*-FFQ is completed and aims to improve a person’s adherence to the Dutch dietary guidelines. The dietary advice is automatically personalised based on the calculated diet quality score and is provided per component. It consists of general healthy diet information and practical advice to improve the intake of each component. Participants are presented their total diet quality score (online Supplementary Fig. S1) and diet quality score per component together with dietary advice (online Supplementary Fig. S2). In addition, the tool presents an overview of results to monitor changes in diet quality over time.

#### Health-related quality of life

HRQoL was assessed with the validated Short Inflammatory Bowel Disease Questionnaire. It consists of ten items each with a 7-point Likert scale resulting in a possible score range of 10–70. Higher scores indicate better HRQoL^(12)^.

#### Clinical disease activity

Clinical disease activity was assessed with the Patient Harvey Bradshaw Index for participants with Crohn’s disease and the Patient Simple Clinical Colitis Activity Index for participants with ulcerative colitis and IBD-unclassified^(13,14)^. Clinical remission was defined as a Patient Harvey Bradshaw Index score ≤4 and a Patient Simple Clinical Colitis Activity Index score ≤2^(15,16)^.

#### Evaluation

Participants’ experiences with the *Eetscore* tool were evaluated with a self-composed evaluation consisting of questions about the *Eetscore*-FFQ and the personalised dietary advice.

### Statistical analysis

Participants who completed baseline and at least one follow-up assessment were included in the analysis. Normally distributed data are presented as mean values and standard deviations, skewed data as median with interquartile range (IQR) and categorical data as counts and percentages.

Linear mixed models were performed to assess changes in diet quality within subjects over time (fixed main factor) and to account for missing values. Baseline values were used as reference. If effect of time was significant, pairwise comparisons between the different time points were conducted with Bonferroni correction to adjust for multiple comparisons.

Multivariate linear regression analysis was performed to identify factors associated with diet quality at baseline. First, the following variables were evaluated in univariable analysis: age, sex, BMI, level of education, smoking behaviour, diagnosis, disease duration and clinical disease activity. Factors with a *P*-value < 0·2 in the univariable analysis, and BMI and clinical disease activity were included in the multivariable analysis. Linear mixed models were performed to identify factors associated with diet quality over time with an identity covariance structure and indicating time as repeated measure. Diet quality was the dependent variable and the same variables as in the regression analysis were added as fixed main effects to the model.

Linear mixed models were performed to assess changes in HRQoL and clinical disease activity within subjects over time (fixed main factor) and to account for missing values. Pairwise comparisons were performed similar to the analysis of diet quality. If HRQoL or clinical disease activity significantly changed over time, further linear mixed model analyses were performed to assess if those changes resulted from changes in diet quality (main fixed covariate) with an identity covariance structure and indicating time as repeated measure. After univariate analysis, the following variables were evaluated as fixed main effects: age, sex, BMI, level of education, biologics as highest step-up in IBD medication and clinical disease activity. Variables with a *P*-value < 0·2 were included in the multivariable analysis.

Linear mixed model data are reported as fixed effect estimates with 95 % confidence intervals. A *P*-value of <0·05 was considered statistically significant. Statistical analysis was performed using IBM SPSS Statistics version 25.0.

## Results

**Fig. 1.**
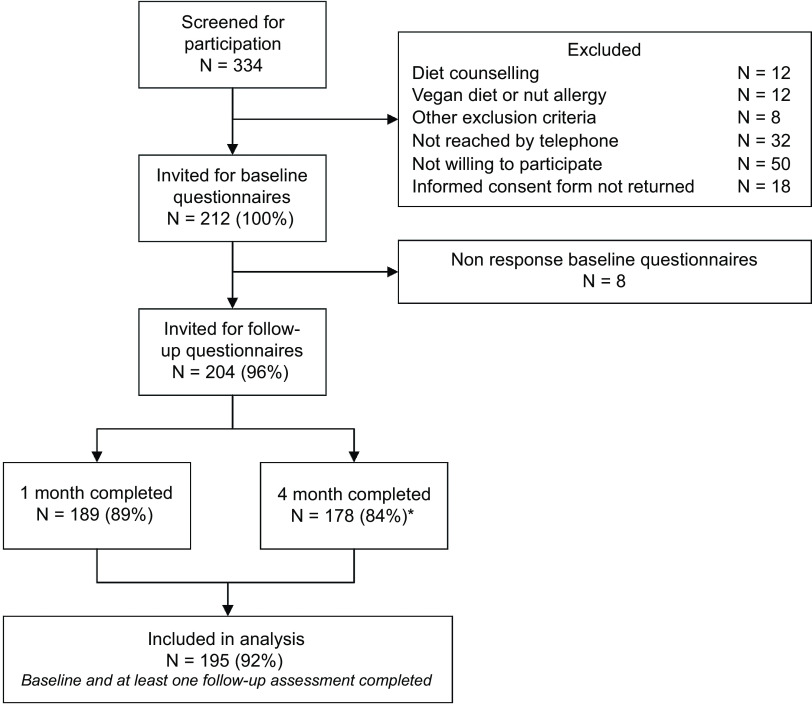
Flowchart of participant screening and inclusion. *At 4-month follow-up, 180 participants completed the evaluation about the *Eetscore*.

In this study, 212 participants were invited for baseline questionnaires (Fig. 1). Participants who completed the baseline questionnaires (*n* 204) were invited for follow-up assessments after 1 and 4 months. In total, 195 participants completed at least one follow-up assessment and were included in the analysis.

Of the 195 participants, 60 % was female, median age was 47 years (IQR 32–57) and BMI was 25 kg/m^2^ (IQR 22–28) (Table 2). The non-responders (*n* 17) were slightly different in terms of sex (41 % female) and age (37 years (27–47)) while BMI was comparable. Median disease duration of participants was 10 years (IQR 4–18) and half of the participants had Crohn’s disease. At baseline, 77 % of participants used IBD medication and 72 % was in clinical remission. In their habitual diet, 28 % of participants applied certain dietary restrictions. Most common restrictions were exclusion or limitation of meat and lactose products. Other frequently mentioned restrictions were exclusion or limitation of gluten, carbohydrates, added sugar and ultra-processed food products.

**Table 2.** Participant characteristics of the total study population (Median values and interquartile ranges; numbers and percentages)

	*n* 195	%
Female	117	60
Age: years		
Median	47	
IQR	32–57	
BMI: kg/m^2^		
Median	25	
IQR	22–28	
Level of education*		
Low	41	21
Intermediate	71	36
High	83	44
Currently smoking	17	8·7
Diagnosis		
CD	97	50
UC	89	46
IBD-U	9	4·6
Disease duration: years		
Median	10	
IQR	3·6–18	
Age at diagnosis CD		
A1 ≤ 16 years	9	9·3
A2 17–40 years	62	64
A3 > 40 years	26	27
Disease localisation CD		
L1 Terminal ileum	25	26
L2 Colon	23	24
L3 Ileocolon	48	49
L4 Upper gastrointestinal	1	1·0
Disease behaviour CD		
B1 Non-structuring, non-penetrating	65	67
B2 Structuring, non-penetrating	25	26
B3 Structuring, penetrating	7	7·2
Perianal disease CD	19	20
Disease extent UC and IBD-U		
E1 Ulcerative proctitis	11	11
E2 Left-sided distal colitis	38	39
E3 Extensive pancolitis	49	50
Extra-intestinal manifestations	33	17
IBD medication currently used†		
None	44	23
5-aminosalicylic acid	71	36
Steroids	19	10
Immunomodulators	52	27
Biologics	58	30
Highest step-up in IBD medication		
5-aminosalicylic acid	22	11
Steroids	31	16
Immunomodulators	58	30
Biologics	83	43
Prior IBD-related surgery	34	17
Clinical disease activity‡		
Remission	140	72
Active disease	55	28
Dietary restrictions†	53	27
Vegetarian or limited meat intake	20	10
Gluten-free or limited gluten intake	5	2·6
Lactose-free or limited lactose intake	14	7·2
Low in carbohydrates	5	2·6
Other	16	8·2

IBD, inflammatory bowel disease; CD, Crohn’s disease; UC, ulcerative colitis; IBD-U, inflammatory bowel disease-unclassified.

* Level of education: no education, primary or lower vocational education and lower general secondary education (low); secondary vocational education and higher general secondary education (intermediate); higher vocational education and university (high).

† Multiple options possible per participant.

‡ Defined by Patient Harvey Bradshaw Index ≤ 4 or Patient Simple Clinical Colitis Activity Index ≤ 2 and expressed as number (%).

Values expressed as number (%), unless stated otherwise.

**Fig. 2.**
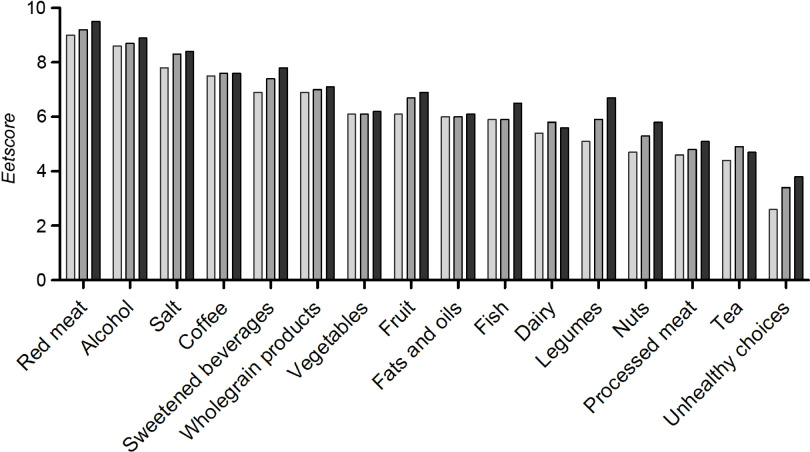
Eetscore per food component at (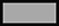) baseline, (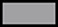) 1 month and (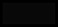) 4 months.

Mean diet quality at baseline was 98 (sd 19) points out of 160 points. Component scores were highest for red meat, alcohol, salt, sweetened beverages and wholegrain products, suggesting that the intake of these food groups was (almost) in line with dietary recommendations (Fig. 2). Component scores were lowest for legumes, nuts, processed meat and unhealthy choices. Median intake per food component in g/d is described in online Supplementary Table S1. As reflected in diet quality scores, participants’ median intake of vegetables, fruit, legumes, nuts, dairy products and fish was lower than recommended, while the median intake of processed meat was higher than recommended.

After using the *Eetscore*, diet quality increased to 103 (sd 21) points after 1 month and 107 (sd 21) after 4 months (Table 3). This increase was significant between baseline and 1 month (*P* < 0·001), baseline and 4 months (*P* < 0·001) and 1 month and 4 months (*P* = 0·005). Biggest improvements were seen in legumes, unhealthy choices, nuts and sweetened beverages (Fig. 2).

**Table 3.** Diet quality, health-related quality of life and clinical disease activity at baseline, 1 month and 4 months (Median values and interquartile ranges; mean values and standard deviations)

	Baseline *n* 195		1 month *n* 189		4 months *n* 178		*P*
	Median	IQR	Median	IQR	Median	IQR	
Diet quality*							
Mean	98		103		107		<0·001
SD	19		21		21		
Health-related quality of life†	54	46–61	56	48–62	56	45–62	0·001
Clinical disease activity‡							
Crohn’s disease	2·5	1·0–4·3	3·0	1·0–5·0	3·0	1·0–5·0	0·88
Ulcerative colitis and IBD-U	1·0	0·0–3·0	1·0	0·0–2·8	1·0	0·0–3·0	0·13

IBD-U, inflammatory bowel disease-unclassified.

* Total mean ± sd *Eetscore*.

† Total median (IQR) short Inflammatory Bowel Disease Questionnaire score.

‡ Total median (IQR) Patient Harvey Bradshaw Index for Crohn’s disease and Patient Simple Clinical Colitis Activity Index for ulcerative colitis and IBD-U.

Diet quality at baseline was positively associated with age, female sex and level of education (Table 4). For example, each 10-year increase in age was associated with a three-point higher diet quality. The same factors were positively associated with diet quality over time. BMI and clinical disease activity were not associated with diet quality.

**Table 4.** Factors associated with diet quality at baseline and with change in diet quality over time (*β*-coefficients and 95 % confidence intervals)

Variable*	Diet quality at baseline			Diet quality over time		
	*β*	95 % CI	*P*	*β*	95 % CI	*P*
Age (in years)	0·31	0·12, 0·50	0·001	0·34	0·16, 0·52	<0·001
Female sex (*v*. male)	9·30	4·04, 14·57	0·001	9·33	4·23, 14·43	<0·001
BMI (kg/m^2^)	– 0·28	–0·85, 0·29	0·34	–0·41	–0·96, 0·14	0·15
Intermediate level of education (*v*. low)	9·05	1·78, 16·31	0·015	9·63	2·60, 16·66	0·008
High level of education (*v*. low)	13·70	6·59, 20·82	<0·001	15·73	8·84, 22·62	<0·001
Clinically active disease (*v*. clinical remission)	–1·21	–7·03, 4·62	0·68	–1·46	–4·52, 1·61	0·35

*R* ^2^ = 15 % for diet quality at baseline.

* Smoking behaviour, diagnosis and disease duration were not associated with diet quality in univariable analysis and therefore not included in the multivariable model.

At baseline, median HRQoL was 54 (IQR 46–61) points (Table 3). HRQoL of participants significantly increased during the study. This increase was only significant between baseline and 1 month (*P* < 0·001). At baseline, median Patient Harvey Bradshaw Index was 2·5 (1·0–4·3) points and median Patient Simple Clinical Colitis Activity Index was 1·0 (0·0–3·0) points. Clinical disease activity did not change during the study.

Linear mixed model analysis showed that each ten-point improvement in diet quality was associated with a 0·5-point increase in HRQoL over time (*β* = 0·5, 95 % CI (0·1, 0·9), *P* = 0·007). This association remained statistically significant in the multivariate model adjusting for age, sex, BMI, clinical disease activity and previous or current use of biologics with each ten-point increase in diet quality resulting in a 0·4-point increase in HRQoL (*β* = 0·4, 95 % CI (0·02, 0·7), *P* = 0·04) (online Supplementary Table S2).

**Fig. 3.**
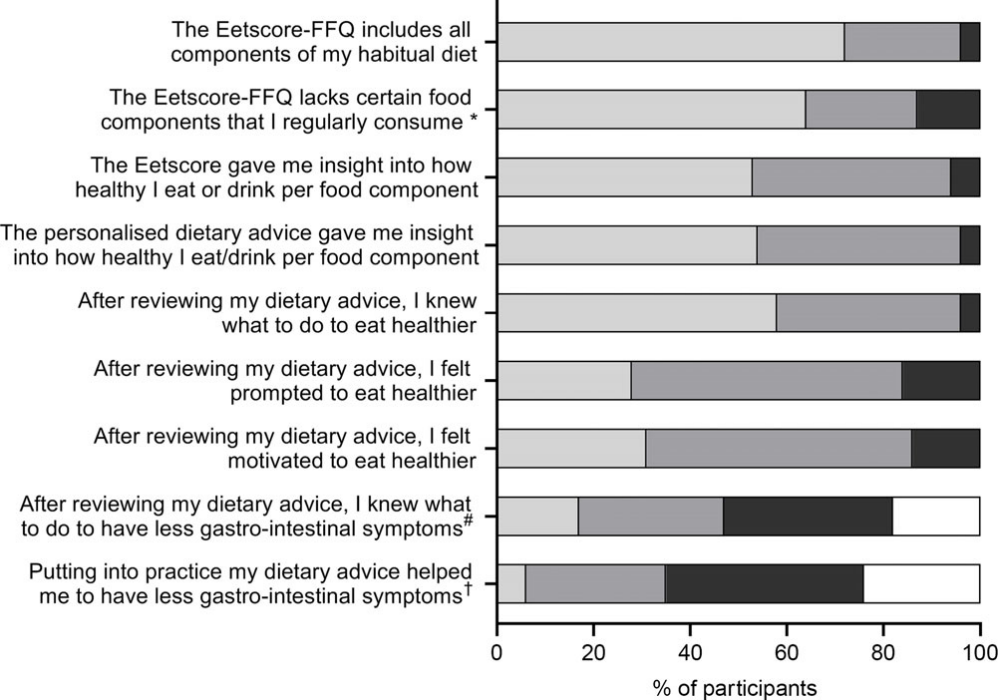
Evaluation of the *Eetscore* tool. *Different answer options: A lot/very well/largely = No, A little = Yes and Not at all = I am not sure. ^#^Most common reasons for choosing ‘other’ were not having gastro-intestinal symptoms during the study, already knowing what to do to have less symptoms or experiencing the advice as too general. ^†^Most common reasons for choosing ‘other’ were not having gastro-intestinal symptoms during the study, not implementing the dietary advice yet or not experiencing an association between diet and gastro-intestinal symptoms in general. 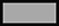, a lot/very well/largely; 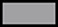, a little; 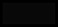, not at all; 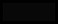, other.

After the last assessment at 4 months, 180 participants completed an evaluation about the *Eetscore* tool (Fig. 3). Of these participants, 23 % reported the *Eetscore*-FFQ lacked regularly consumed food components such as eggs, and lactose-free and soya products. Also, 55 % did not eat or drink all food components of the *Eetscore*-FFQ given allergies, intolerances or dietary restrictions (28 %), personal taste (52 %) or gastrointestinal symptoms (40 %). Almost all participants (96 %, *n* 173) had reviewed their *Eetscore* results of which 92 % believed these results gave insight into how healthy their intake was per food component.

Most participants (93 %, *n* 167) had reviewed the personalised dietary advice of the *Eetscore* (Fig. 3). After reviewing their dietary advice, 96 % of participants knew what to do to eat healthier. Although the primary aim of the advice was to improve diet quality, 47 % of participants also believed to know what to do to have less gastrointestinal symptoms. Putting the dietary advice into practice helped 35 % of participants to have less symptoms. To eat healthier, 65 % would like to receive additional information such as practical advice with examples (22 %), healthy recipes (35 %), explanation about why certain food is healthy (23 %) or information about portion sizes (19 %).

## Discussion

In this study, we used the web-based *Eetscore* tool to assess diet quality of IBD patients and to provide personalised dietary advice supporting a healthy diet. At baseline, intake of red meat, wholegrain products and sweetened beverages was close to the Dutch dietary guidelines, while intake of dairy products, legumes, nuts and processed meat was not. Diet quality significantly improved following personalised dietary advice. IBD patients who were older, female and higher educated had a better diet quality over time. Improvement of diet quality was associated with a small improvement in HRQoL. Clinical disease activity did not change. The *Eetscore* can provide IBD patients insight into how healthy their habitual diet is and what to do to better adhere to the Dutch dietary guidelines.

To date, the current *Eetscore*-FFQ has only been used once in a general, healthy population cohort of 751 Dutch participants^(10)^. Compared with this general cohort, diet quality was lower in our IBD cohort (mean 98 (sd 19) *v*. 111 (sd 18)) with lower scores on fruit, legumes, processed meat, sweetened beverages and unhealthy choices. This may be explained by differences in characteristics of the study populations. Many of their participants were highly educated (65 %), few smoked (6 %) and few were overweight or obese (27 % and 8 %) indicating a health-conscious population. In line with our study, older age, female sex and higher level of education were positively associated with diet quality^(10)^. Using the web-based *Eetscore*, diet quality of participants improved by ten points (i.e. 6 % out of a maximum of 160 points). This better adherence to the Dutch dietary guidelines has been associated with a lower all-cause mortality and a reduction in the risk of stroke, depression and colorectal cancer^(17)^.

Consistent with our findings, previous studies in IBD patients also reported a consumption of dairy products and nuts^(18–20)^ below the recommendations, an overconsumption of processed meat and an adequate consumption of red meat^(18)^. In contrast to our results, previous studies in IBD patients indicate a sufficient intake of legumes^(18,19)^. IBD patients often avoid lactose products and legumes as they believe this prevents abdominal pain, bloating and disease flares, which may explain the low dairy products and legume intake^(3,21)^. In our study, 7 % of participants reported to consume little or no lactose products and while they may have compensated for this with alternatives such as soya milk, this cannot be entered in the *Eetscore*-FFQ. The reason for a nut consumption below the recommendations is unclear. In our study, participants did not report specific reasons for avoiding nuts and those allergic to nuts were excluded.

A recent study showed that long-term dietary patterns enriched with legumes, vegetables, fruits and nuts, more plant-based instead of animal-based products and avoidance of alcoholic beverages, high-fat processed meats and soft drinks have the potential to prevent intestinal inflammatory processes via the gut microbiome^(22)^. This potentially favourable dietary pattern for IBD patients is in line with the Dutch dietary guidelines and the *Eetscore* can provide insight in adherence to these guidelines. Moreover, IBD patients consider dietary guidance to be important, while only a minority feels to have received adequate information from their physician^(23)^. The *Eetscore* can fulfil this need for dietary guidance of IBD patients.

Several studies investigated dietary patterns and their association with HRQoL and clinical disease activity^(18,24,25)^. A Western dietary pattern characterised by the intake of refined grains, red and processed meat, condiments and sauces, and unhealthy choices was associated with flare occurrence^(18)^, while adherence to a Mediterranean dietary pattern characterised by a high consumption of vegetables, fruit, wholegrains and nuts, and a low consumption of red and processed meat and processed foods was associated with a higher HRQoL and lower disease activity^(24,25)^. Our observations support that better adherence to the Dutch dietary guidelines, which share characteristics with the Mediterranean diet, is associated with improved HRQoL. In contrast to previous studies, we did not find an association between diet quality and clinical disease activity. This might be explained by the relatively short follow-up time and low number of participants with clinically active disease.

Relatively few participants reported that use of the *Eetscore* tool helped to reduce their gastrointestinal symptoms. During this study, the majority of participants did not experience gastrointestinal symptoms. Besides, many participants commented to already know very well which foods work best for them to reduce or prevent gastrointestinal symptoms after several years of living with IBD. Also, the *Eetscore* tool is not designed to help reduce gastrointestinal symptoms. For example, IBD patients often avoid certain food components because of abdominal complaints and the *Eetscore* does not provide alternatives (e.g. for dairy products or legumes)^(26)^. Dietary advice of the *Eetscore* may be optimised for IBD patients by suggesting alternative products to achieve a better diet quality while limiting abdominal complaints.

The main strength of this study is its prospective nature with repeated dietary assessment and advice in a clinical setting using the *Eetscore.* In addition, the *Eetscore* is a quick and easy tool to assess diet quality compared with commonly used extensive FFQ. Besides, we included a large, representative cohort of IBD patients with varying ages and different disease phenotypes. Finally, the response rate was very high (92 %), thereby limiting the risk of non-response bias. However, this study was limited by the absence of a control group. Therefore, we cannot conclude whether diet quality improved as a result of the personalised dietary advice of the *Eetscore* or as a result of increased dietary awareness. In line with this, studies on lifestyle factors are prone to socially desirable answers, which may have led to an overestimation of diet quality. However, participation was anonymous and results were not shared with participants’ gastroenterologist or IBD nurse. Also, completing the *Eetscore* several times may have induced a learning curve with participants being aware of the correct answers when completing the *Eetscore* for the second or third time. However, a previous study showed no change in diet quality when the *Eetscore*-FFQ was completed twice within 4 months in the absence of dietary advice^(10)^. Additionally, memory bias and estimation error may have occurred as these are inherent to using FFQ^(27)^. Lastly, the *Eetscore*-FFQ covers approximately 85 % of dietary intake, is based on regularly consumed food products and does therefore not assess plant-based alternatives of meat and dairy products^(8,9)^.

In conclusion, web-based dietary assessment and advice helps IBD patients to improve their diet quality. The *Eetscore* tool is easy to use, gives practical insight into dietary intake and supports a healthy diet in IBD patients. It may be further optimised by adding healthy alternatives for food products that are commonly avoided by IBD patients.

## Supplementary material

For supplementary material accompanying this paper visit https://doi.org/10.1017/S0007114522001064. click here to view supplementary material
