# Supplementary material for: Web-based dietary assessment and advice helps inflammatory bowel disease patients to improve their diet quality
Source: Br J Nutr. 2022 Apr 4;129(2):231–9. doi: 10.1017/S0007114522001064 (PMC9870719; doi:10.1017/S0007114522001064)
Supplement: Supplementary file 1 [file S0007114522001064sup001.docx]

**Supplementary Tables**

**Supplementary Table 1 Intake of relevant food components in grams per day**

|  | **Intake at baseline**  **N = 195** | **Recommended intake** |
| --- | --- | --- |
| Vegetables | 126 [72 – 183] g/day | ≥ 200 g/day |
| Fruit | 139 [59 – 176] g/day | ≥ 200 g/day |
| Wholegrain products | 129 [87 – 189] g/day | ≥ 90 g/day |
| Legumes | 4.4 [0.0 – 17] g/day | ≥ 10 g/day |
| Nuts | 4.3 [1.4 – 16] g/day | ≥ 15 g/day |
| Dairy* | 169 [84 – 316] g/day | 300-450 g/day |
| Fish | 9.6 [5.8 – 15] g/day | ≥ 15 g/day |
| Red meat | 22 [7.2 – 43] g/day | ≤ 45 g/day |
| Processed meat | 25 [9.5 – 49] g/day | 0 g/day |

Values expressed as median [IQR]. *Including milk, yoghurt and cheese products.

**Supplementary Table 2 Factors associated with change in health-related quality of life over time**

| **Variable *** | **β** | **95% CI** | **P-value** |
| --- | --- | --- | --- |
| Diet quality *(*total *Eetscore)* | 0.04 | 0.002; 0.07 | **0.04** |
| Age (*in years)* | 0.09 | 0.01; 0.17 | **0.02** |
| Female gender *(vs. male)* | – 2.79 | – 5.02; – 0.56 | **0.02** |
| BMI (*kg/m^2^*) | – 0.43 | – 0.67; – 0.19 | **< 0.001** |
| Biologics as highest step-up | – 1.28 | – 3.51; 0.95 | 0.26 |
| Clinically active disease *(vs. clinical remission)* | – 6.58 | – 5.85; – 5.32 | **< 0.001** |

Bold values are significant. * Level of education was not associated with health-related quality of life in univariable analysis and therefore not included in the multivariable model. **Supplementary Figures**

**Supplementary Figure 1 Diet quality score of an individual participant as shown in the *Eetscore* tool**

**
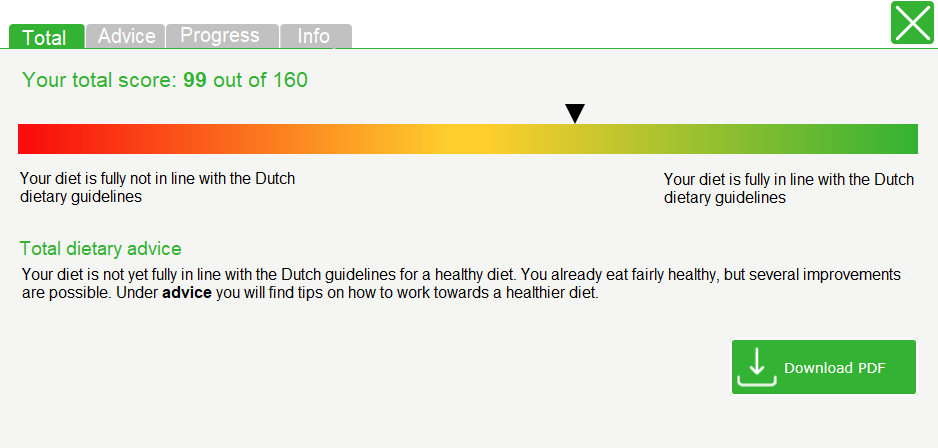
**

**Supplementary Figure 2 Personalised dietary advice of an individual participant as shown in the *Eetscore* tool**

**
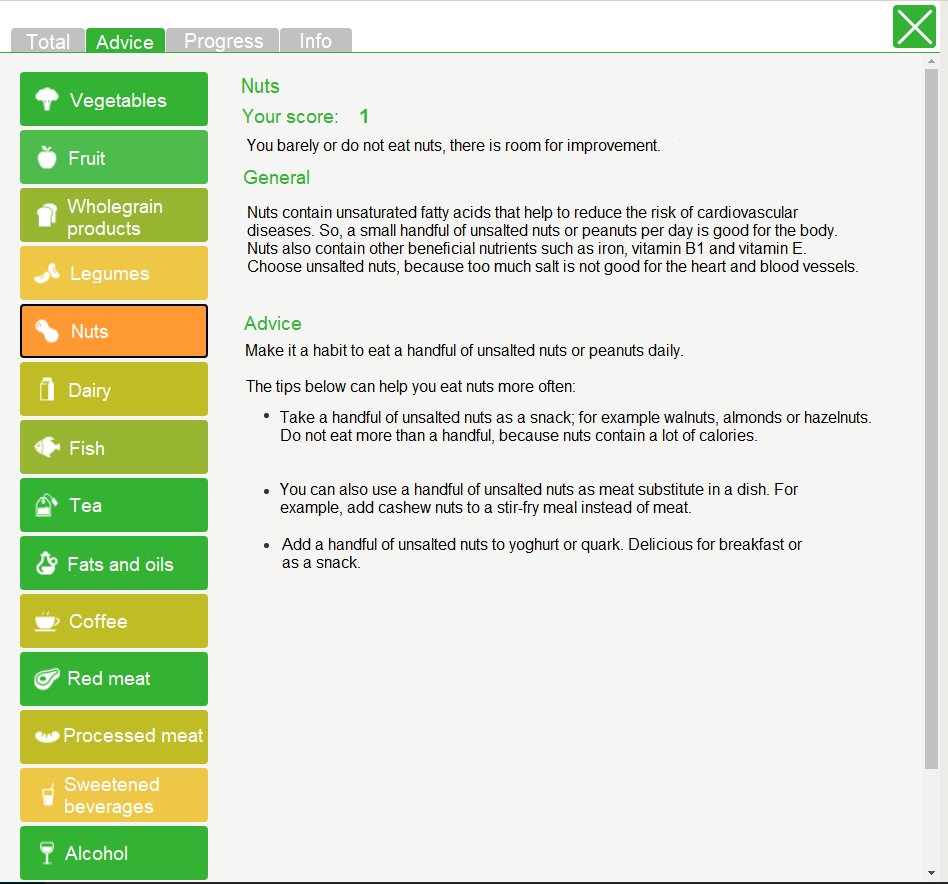
**
